# Insights into pain distraction and the impact of pain catastrophizing on pain perception during different types of distraction tasks

**DOI:** 10.3389/fpain.2024.1266974

**Published:** 2024-01-23

**Authors:** Arash Asefi Rad, Pia-Maria Wippert

**Affiliations:** ^1^ Medical Sociology and Psychobiology, Department of Health and Physical Activity, University of Potsdam, Potsdam, Germany; ^2^ Faculty of Health Sciences, Joint Faculty of the University of Potsdam, Brandenburg Medical School Theodor Fontane, and the Brandenburg University of Technology Cottbus-Senftenberg, Postdam, Germany

**Keywords:** pain modulation, experimental pain, neuromodulation of pain, pain intensity, pain unpleasantness, pain catastrophizing

## Abstract

**Introduction:**

Distraction is commonly used to reduce pain, but the effectiveness of distractions remains inconclusive. Studies have shown that pain catastrophizing could modulate the effectiveness of distraction strategies. The present study aimed to compare various distraction tasks, then control for pain catastrophizing, and examine how this relationship varies with pain intensity and unpleasantness across different distraction tasks.

**Methods:**

Forty-one pain-free participants (aged 27.00 ± 5.41) were recruited for a cross-sectional study. Four types of distraction (cognitive, sensory, emotional, and social) were presented, while moderate pain intensity was induced by electrical stimulation. Before starting the experiment, moderate pain intensity was individually calibrated as six on the Numerical Pain Rating Scale (NRS) to control individual differences in pain sensitivity. Each participant performed all four distraction tasks in a random order. NRS measured pain assessment. Pain catastrophizing was measured by the Pain Catastrophizing Scale (PCS). A repeated measure ANCOVA was conducted to examine the effects of pain dimensions during distraction tasks as a within-subject and pain catastrophizing as a covariate factor.

**Results:**

A significant difference was observed in the pain intensity and unpleasantness during cognitive distraction. After controlling for PCS, there were diverse associations between PCS and pain intensity across distinct distraction tasks: social vs. sensory, and cognitive vs. sensory distraction. A consistent pattern in pain unpleasantness emerged with minor variations. This interaction underscored notable distinctions between social vs. sensory and emotional distractions, as well as between cognitive vs. sensory and emotional distractions. However, only the correlation in social distraction remained significant in both pain dimensions.

**Discussion:**

Our findings reveal that the link between PCS and pain dimensions varies across different distraction tasks, suggesting diverse interactions. Particularly, social distraction, characterized by both emotional and cognitive states, proves beneficial with lower PCS scores; however, this advantage diminishes as PCS scores increase.

## 1 Introduction

Pain is an unpleasant sensory and emotional experience that is triggered by physical, social, or emotional circumstances, with its intensity of perception contingent on an individual's cognitive appraisal (1). This complex phenomenon can be evaluated in two dimensions: the sensory dimension, which reflects the intensity of pain, and the affective dimension, which measures the unpleasantness of pain (2, 3). In the past, the pain domain has traditionally focused on biological factors, but this narrow approach fails to explain why individuals may perceive pain disparately despite similar nociceptive stimuli. Later, an interdisciplinary approach called the biopsychosocial model was developed, emphasizing the dynamic interplay between physical, psychological, and socio-environmental factors to achieve a holistic understanding of pain and its treatment (4, 5). Therefore, psychological factors are not merely a consequence of pain but also impact all stages of pain (4, 6). Incorporating these factors can enhance the understanding of pain and ultimately improve pain management strategies (1).

Distraction is a common noninvasive strategy for coping with pain used in various ways. The aim is to divert attention away from noxious stimuli (7–10). In this process, pain signals still exist, but the individual's attention is on a more demanding task. Although several studies have examined the efficacy of distraction techniques in comparable pain settings and age cohorts, results have been ambiguous. Some studies have reported pain relief (6, 8, 11, 12), while others have found no effects (13–16), and there have even been instances of increased pain perception (6, 17). The conflicting outcomes can be partly attributed to the type of distraction tasks (6). Distractions use a wide range of tasks mainly based on cognitive state, emotional state, or a combination of both. In the cognitive state, the distractor stimulus competes with pain for limited attentional resources. As a result, more capacity is provided for attentional allocation to other stimuli as distractor tasks, causing decreased pain perception (18–20). Through this kind of distraction task, this stimulus focuses on the processes and structures of working memory in a temporary time frame to attain a specific goal (20–22). The emotional state contains three dimensions, pleasure (the pleasantness of a stimulus), arousal (the intensity of a stimulus), and dominance (feeling of control) (23). This state can alter pain perception via auditory or visual tasks through positive or negative emotions; positive emotions can reduce pain perception, while negative emotions can increase it (6, 10). Studies showed that pleasure and arousal dimensions interact with each other in altering pain perception (10, 24). Brain imaging studies showed that cognitive and emotional states change afferent pain pathway activities and activate different systems in the brain. These areas mainly include the primary somatosensory cortex (S1), the insula for the cognitive state, and the anterior cingulate cortex (ACC) for the emotional one (2, 25). Moreover, stress and pain stimuli are processed in similar regions in the brain (25). Adding the emotional component to the cognitive state of distraction might have more pronounced effects on altering pain perception (6) due to the evoking of multiple brain regions by pain stimulation, emotional regulation, and stress and anxiety reduction (25, 26). Sensory distraction (13, 27) and social interaction (28) are examples of these distractor tasks.

In addition to the distraction strategies, the psychosocial component, specifically pain catastrophizing, plays a decisive role in the pain management process. Pain catastrophizing involves magnifying pain's perceived threat and negative consequences, leading to heightened distress and negative emotions. It includes an excessive focus on pain, rumination, and expecting the worst outcomes (29). It is positively associated with pain perception, and can account for 7%–31% of the variance in pain perception depending on the type of pain and the characteristics of the population (15, 29, 30). A comprehensive understanding of pain catastrophizing has the potential to enhance intervention strategies.

Three similar theoretical models, *schema activation*, *threat appraisal*, and *attentional models*, have been proposed to understand the relationship between pain outcome and catastrophizing (9, 15, 30, 31). *Schema activation* involves connecting the present sensory stimulus to the relevant schema (pain schema) from memory. The sensory pain information is processed simultaneously and parallels with the emotional system, and their interaction could reconstruct pain perception. This integration is shaped based on schematic processing, a cognitive framework that helps organize and interpret information (1, 30–33). The *threat appraisal model* explains the evaluation of pain catastrophizing, which causes a specific response to the stimulation as an appraisal (1, 30, 34). Appraisal judgment of pain does not allow the individual to get distracted from pain stimuli. The first two models describe the development of thoughts and beliefs about pain according to the schema or threat information (1). Eventually, the *attentional model* links pain catastrophizing to the physiological pain aspects, intensifying their preferential and dysfunctional attention to pain-related information (1, 30). The more severe pain is perceived, the more this stimulus occupies the attentional resources. As a result, this process affects cognitive coping pain strategies, such as diverting attention from pain and ignoring it (34). However, these three models should not be considered mutually exclusive because they share conceptual overlap, and each can describe a different domain of the catastrophizing pain mechanism.

Studies have indicated that the impact of distraction on pain perception can be variable (1), and individuals with high levels of pain catastrophizing may be less responsive to distracting tasks and more likely to stay engaged in the pain process (30, 35). Prins et al. conducted a study comparing mindfulness and distraction groups in undergraduate students experiencing heat pain. Participants were assigned to either the mindfulness group, where they listened to a mindfulness instruction, or the distraction group, where they listened to a prerecorded story, throughout the pain exposure. They proposed that the selection of a distraction or mindfulness strategy may depend on the individual's level of pain catastrophizing to achieve the greatest benefit. Notably, when pain catastrophizing was high, pain was less perceptible in the mindfulness group than in the distraction group, while the opposite effect was observed when the level of pain catastrophizing was low (9). A recent study by Rischer et al. reported that individuals with average or high levels of pain catastrophizing experienced more pain reduction during distraction tasks, but only if they had better sustained attention abilities. This effect was not observed in individuals with low levels of pain catastrophizing (36).

Distraction tasks, varying in cognitive, emotional, or combined states, possess diverse qualities. These tasks can be influenced by individual factors, impacting their effectiveness. The relationship between distraction quality and pain perception in individuals with pain catastrophizing remains unclear. To address this gap, a study was conducted on healthy volunteers with experimental pain induction to minimize confounding factors such as using analgesic drugs and physiological and psychological biases (37). Considering the potential effects of distraction tasks on pain perception and the influence of dispositional pain catastrophizing levels, we formulate three research questions: (1) Are there differences between distraction tasks in pain intensity and unpleasantness? (2) Are there differences between distraction tasks in pain intensity and unpleasantness when controlling for the influence of pain catastrophizing? (3) Does the relationship between pain catastrophizing and pain perception consistent across distraction tasks?

## 2 Materials and methods

### 2.1 Participants

41 pain-free participants (aged 18–65 years) were recruited by personal contact or through flyer advertising at Potsdam University. Exclusion criteria were: feeling of acute or chronic pain, long-term pain medication, pregnancy, cuts or sores in the non-dominant hand, and a history of self-reported neurological or cardiovascular disease. Participants had to avoid any analgesics (48 h) and alcoholic beverages (12 h) before the test session. Each participant gave written informed consent. The study was approved by the ethics committee of the University of Potsdam (No. 47/2016 and 36/2011).

### 2.2 Design and procedure

This cross-sectional study included four different distraction tasks. Each distraction task was assigned to one block. To complete the whole experiment, participants had to finish four blocks.

**Figure 1:**
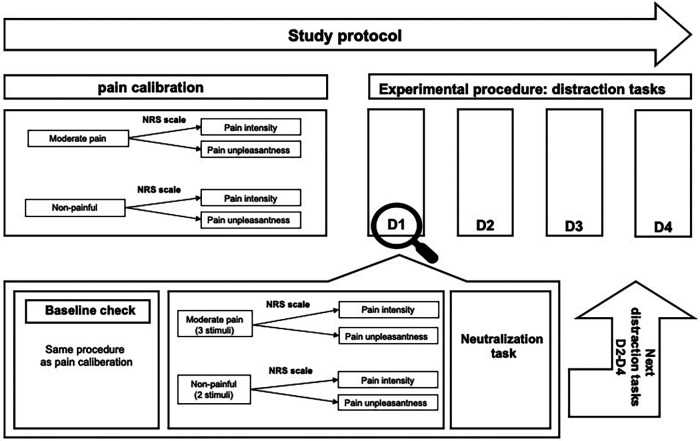
Top: schematic representation of the study protocol. D1–D4 represent randomized distraction tasks, and each distraction task shows one block. Bottom: overview of one experimental block. In total, participants received four experimental blocks, one for each distraction task. D1–D4: different distraction tasks, NRS, numerical pain rating scale.

After screening the inclusion and exclusion criteria, participants were asked to rate their pain intensity in the last 24 h on a Visual Analog Scale (VAS) and Chronic Pain Grade (CPG). The VAS was a 100 mm continuum line anchored from no pain to extremely intensive pain (38). The CPG is a questionnaire that grades two dimensions of chronic pain severity over the past six months: Characteristic pain intensity (CPI) and subjective pain disability (DISS). It consists of seven items, and each dimension score is from 0 (no pain) to 100 (severe pain) CPG points (39). The aim was to eliminate the influence of acute or chronic pain on participants' pain perception during the experiment. Participants also completed the German version of the Pain Catastrophizing Scale (PCS) (40). Afterwards, individual pain calibration was conducted to ensure a consistent administration of moderate and non-painful stimuli among all participants. The experiment began with the first block of the distraction task, wherein a neutralization task was performed between each block to minimize any potential carryover effects from previous distractions (Figure 1). For this purpose, participants were requested to count backward a three-digit number (e.g., 790) for a minute, which was the same for all participants. Participants were tested individually by two trained test leaders. One test leader was responsible for controlling the electrical pain stimuli while hidden behind a partition. The other test leader guided the participant through the experiment. The entire data collection process lasted for a total of 90 min per participant.

### 2.3 Pain stimuli and calibration

A constant current stimulator induced pain through electrostimulation for 250 ms (electrical stimulator D7SA, Digitimer), that was connected to a pair of Ag/AgCl electrodes. The electrodes were attached to the upper and lower surface of the volar forearm of the participant's non-dominant hand.

For pain calibration, the intensity of electrostimulation started at 0 mA with an increasing intensity of 0.5 mA until the participant reported non-painful and moderate pain, in accordance with an 11-point Numerical Rating Scale (NRS). Non-painful and moderate pain were defined as two and six on the NRS, respectively (41). Once the respective pain levels were reached, the electrical intensities were noted and used during the further experiment.

In each experimental block, a series of 5 trials took place. Each trial involved the presentation of a single noxious stimulus. The set of 5 stimuli within a block included 3 moderate pain stimuli and 2 non-painful stimuli, with their randomized order. For instance, the first block could be structured as follows: moderate pain, moderate pain, non-painful, moderate pain, and non-painful. Non-painful stimulus was used to minimize pain stimulus habituation and was not considered for calculation (42, 43). Altogether, the experiment included four blocks of distraction tasks, twelve moderate and 8 non-painful stimuli. After each pain stimulus, participants were asked to rate the pain intensity and unpleasantness on an NRS. Participants were informed that they would receive electrical pain stimuli but were unaware of the pain protocol.

### 2.4 Distraction tasks

Four distraction techniques (cognitive, emotional, sensory, and social distraction) were used in a randomized order. To ensure a uniform procedure for participants, cognitive, emotional, and sensory distraction tasks were recorded and played back by Psychopy software (version 1.83) (44). Social distraction was performed by one of the test leaders. Each distraction task constituted one block, and each block comprised 5 trials. The duration of each trial was less than 1 min.

#### 2.4.1 Cognitive distraction

The cognitive distraction task included a 3-back task. This task could affect pain perception by engaging working memory to compete with pain stimuli. It included numbers or colors as items to present in a sequence. Participants had to determine whether the current item matched the item presented three steps earlier in the sequence (22, 25, 43, 45).

#### 2.4.2 Emotional distraction

The emotional distraction (46) was operationalized by a positive exposure tone from the standardized database of the International Affective Digitized Sounds (IADS) (23, 47). These normative sounds survey emotion and attention in the experimental investigation. In this study, participants listened to a pool of 5 sounds that were randomly arranged.

#### 2.4.3 Sensory distraction

A short revised version of a body scan which is part of most Mindfulness-Based Stress Reduction (MBSR) programs was chosen as a sensory distraction (25). Participants were asked to actively concentrate on the body's pain-free areas while being relaxed in order to stress reduction.

#### 2.4.4 Social distraction

The social distraction was done based on the interaction between the participant and one of the test leaders. The questions referred to the participant's daily life (e.g., How long did it take you to come to the test? How was the traffic today?) (25).

### 2.5 Outcome variables and instruments

The NRS is an 11-point scale ranging from 0 (no pain) to 10 (extremely intense pain) assessing pain sensory (intensity) and affective pain quality (unpleasantness) through distraction tasks and baseline (38).

The PCS a self-report retrospective measurement that assesses feeling and thoughts of the person while he is in pain. It includes three subscales which are entitled rumination (4 items), magnification (3 items), and helplessness (6 items) in the face of pain. In total, this questionnaire consists of 13 items rated on a Likert scale from 0 to 4 (not at all—all the time), which ranges from 0 to 52. A higher score indicating a higher catastrophizing of pain (48). Cronbach's alpha was 0.91.

Furthermore, the study gathered sociodemographic data, alcohol and nicotine consumption information, medication usage, and participants' pain levels using the Brief Pain Inventory (BPI) (49) over a 24-h period, for a detailed description of the participant characteristic.

### 2.6 Data analysis

Both dimensions of pain—intensity and unpleasantness—were evaluated by calculating means from three moderate pain trials in each block, with each block representing one of the distraction tasks. Descriptive statistics, including means and standard deviations for pain intensity and unpleasantness in each distraction task, were reported during both baseline and distraction. For inferential analysis, changes in pain intensity (baseline—after distraction) and unpleasantness (baseline—after distraction) were examined as dependent variables using repeated-measures ANCOVA. Distraction tasks, covering cognitive, sensory, emotional, and social strategies, were treated as within-subject factors. Pain catastrophizing served as a time-invariant covariate factor, varying only between subjects.

In the subsequent analysis, contrasts and pairwise comparisons were conducted for follow-up. Ultimately, a correlation analysis was performed to evaluate how the covariate contributed to the variation in pain perception during distraction compared to the baseline across the various levels of the within-subjects factor involving four distinct distraction tasks. The level of significance was set at *α* = 0.05. All statistical analyses were performed with IBM SPSS Statistics, version 25 (IBM Corporation©, Armonk, New York, USA).

## 3 Results

**Figure 2:**
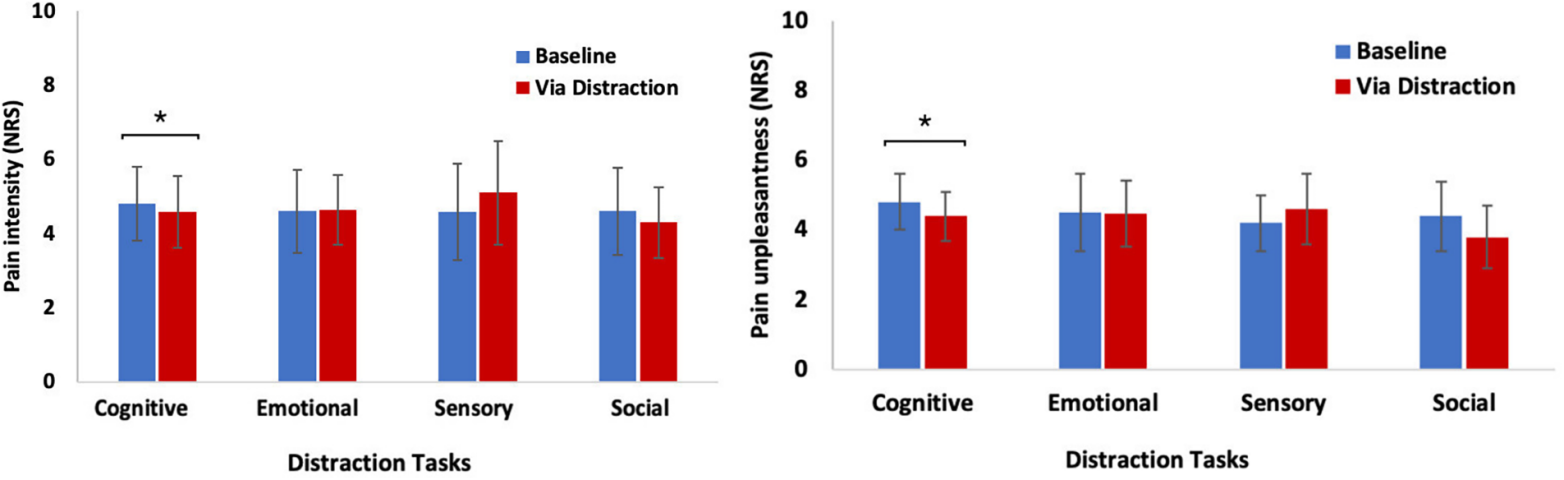
Mean ± standard deviation of pain intensity and unpleasantness before and during each distraction task. *p < 0.05.

A total of 41 participants were recruited for the experiment, out of which 30 (age: Mean (SD) = 27.0 (5.4) years, ≈ 60% female) completed the study. The remaining 11 participants were excluded from further analysis for various reasons: four participants did not appear on the day of the experiment, another four violated the inclusion criteria, and three participants experienced technical problems during the study. The range of PCS was between 0 and 25 points, with an average of 10.20 (SD = 7.77). Approximately, 70% of the sample population was unmarried. In terms of educational attainment, half of the participants were students, while 30% possessed academic degrees. Furthermore, 16% of the participants graduated from technical schools, and 3% did not provide information on their education. With respect to monthly income, 60% of the sample reported earning less than 1,250 euros per month, whereas 13% reported earning between 1,250 and 1,449 euros. Additionally, 13% of the sample reported earnings above 2,250 euros per month. The remaining 14% did not disclose their salaries. Regarding lifestyle habits, the study revealed that nine participants reported regular alcohol consumption, and five participants identified themselves as smokers. Table 1 presents the demographic and psychometric characteristics of the study sample. A Shapiro–Wilk test indicated normal distribution for both pain dimensions in all distraction tasks, except for pain intensity in the sensory task, which was not normally distributed [D(30) = 0.92, *p* = 0.03]. Figure 2 displays the levels of pain intensity and unpleasantness before and during each distraction task.

**Table 1:** Demographic and psychometric characteristics of the sample.

Variables	Total sample (mean ± SD)	Range
Age (years)	27.00 ± 5.41	18–34
Brief Pain Inventory (BPI)	0.21 ± 0.35	0.00 ± 1.14
Current intensity (moderate pain, mA)	4.62 ± 5.35	1–29
Current intensity (neutral pain, mA)	1.52 ± 1.27	0.5–5
CPG: disability (0–100)	5.8 ± 9.9	0–13
CPG: characteristic pain intensity (0–100)	18.5 ± 13.3	0–26.33
PCS-total	10.20 ± 7.77	0–25
• Rumination	4.57 ± 3.70	0–11
• Magnification	2.47 ± 2.06	0–8
• Helplessness	3.17 ± 3.28	0–11

VAS, visual analogue scale; BPI, brief pain inventory related to pain intensity last 24 h; CPG, chronic pain grade; PCS, pain catastrophizing scale.

### 3.1 Pain intensity

Distraction tasks: Results from repeated measures analyses of variance, controlling for age and gender, revealed a significant main effect for the change in pain intensity during cognitive distraction [F(1, 25) = 11.48, *p* = .002, ηp^2^ = .31]. This indicates that participants reported lower levels of pain intensity when engaged in cognitive distraction compared to when no distraction was present.

Controlling the PCS during different distractions: Mauchly's test confirmed the assumption of sphericity [*χ*^2^(5) = 2.20, *p* = .82]. A significant interaction of distraction tasks on pain intensity was observed after controlling for the PCS effect [F(3, 84) = 4.74, *p* = .004, ηp^2^ = .14]. However, subsequent pairwise comparisons did not reveal significant differences in pain intensity among specific distraction tasks when pain catastrophizing was controlled.

**Figure 3:**
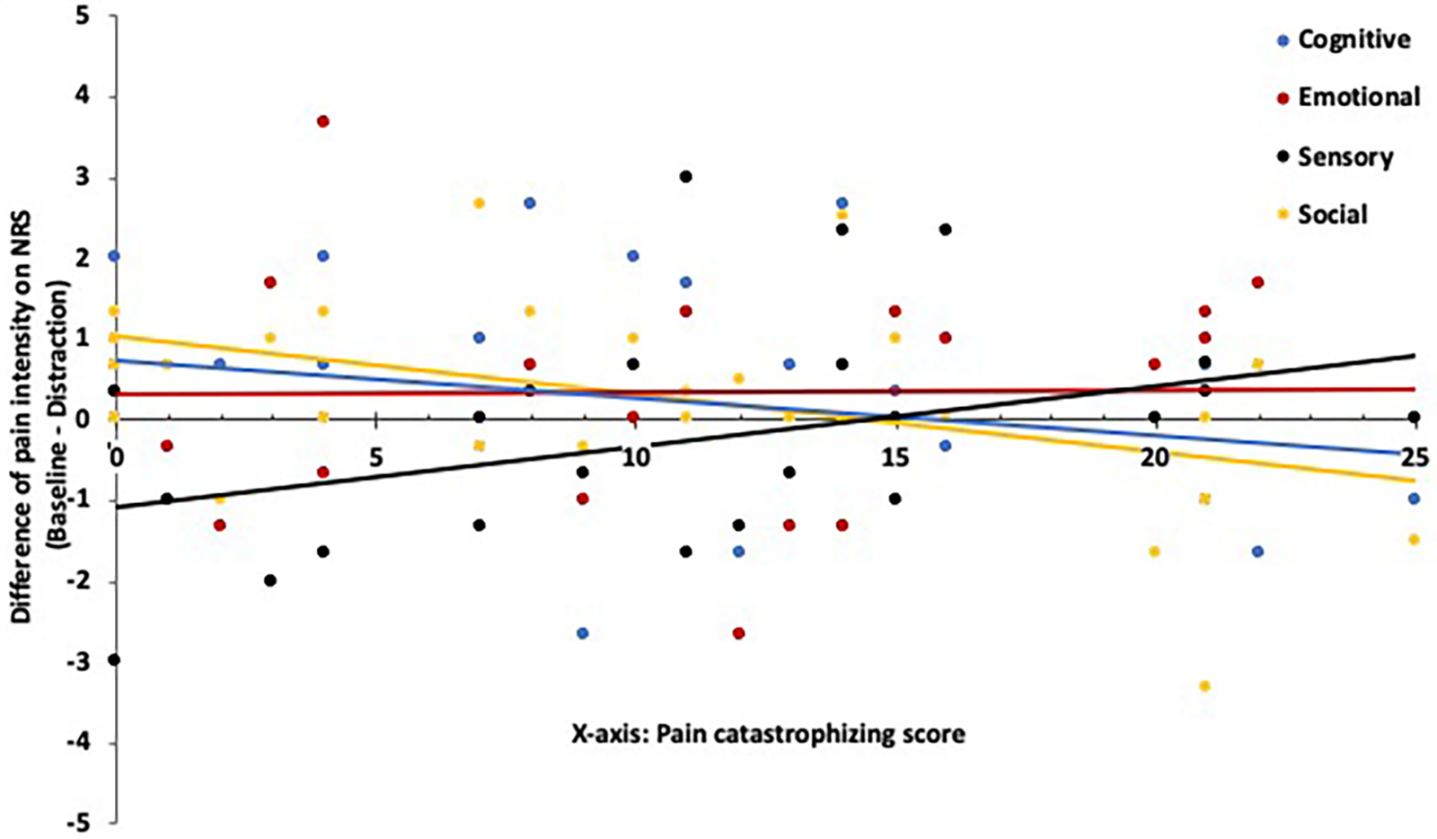
Visual presentation of the PCS's contribution to the change of pain intensity in NRS scale depended on the determined distraction task and respective baseline. A positive score on the y-axis indicates a reduction in pain intensity during distraction, whereas a negative score suggests an increase.

Association between PCS and pain intensity during different distractions: Further analysis revealed significant slope differences in the relationship between pain intensity and PCS score for social compared to sensory distraction [F(1, 28) = 12.05, *p* = .002, ηp^2^ = .30] and cognitive vs. sensory distraction [F(1, 28) = 7.56, *p* = .010, ηp^2^ = .21]. Differences between the other distraction tasks were not statistically significant. Figure 3 illustrates the interaction plot, demonstrating the variation in the relationship between PCS and pain intensity across different levels of distraction tasks. The correlations of changing pain intensity and PCS in each distraction task were as follows: Sensory distraction [*r*(30) = .33, *p* = .07], cognitive [*r*(30) = −.26, *p *= .16], social [*r*(30) = −.46, *p* = .01], and emotional distraction [*r*(30) = .02, *p* = .92]. Only the correlation in social distraction was significant, suggesting a diverse effect where an increase in PCS score led to a smaller reduction or even an increase in pain intensity during this distraction task.

### 3.2 Pain unpleasantness

Distraction tasks: Results from repeated measures analyses of variance, controlling for age and gender, revealed a significant main effect for the change in pain unpleasantness during cognitive distraction [F(1, 25) = 7.09, *p* = .01, ηp^2^ = .22]. This indicates that participants reported lower levels of pain unpleasantness when engaged in cognitive distraction compared to when no distraction was present.

Controlling the PCS during different distractions: Mauchly's test confirmed the assumption of sphericity (*χ*^2^(5) = 10.88, *p* = .054). A significant interaction of distraction tasks on pain unpleasantness was observed after controlling for the PCS effect [F(3, 84) = 6.34, *p* = .001, ηp2 = .18]. However, subsequent pairwise comparisons did not reveal significant differences in pain unpleasantness among specific distraction tasks when pain catastrophizing was controlled.

**Figure 4:**
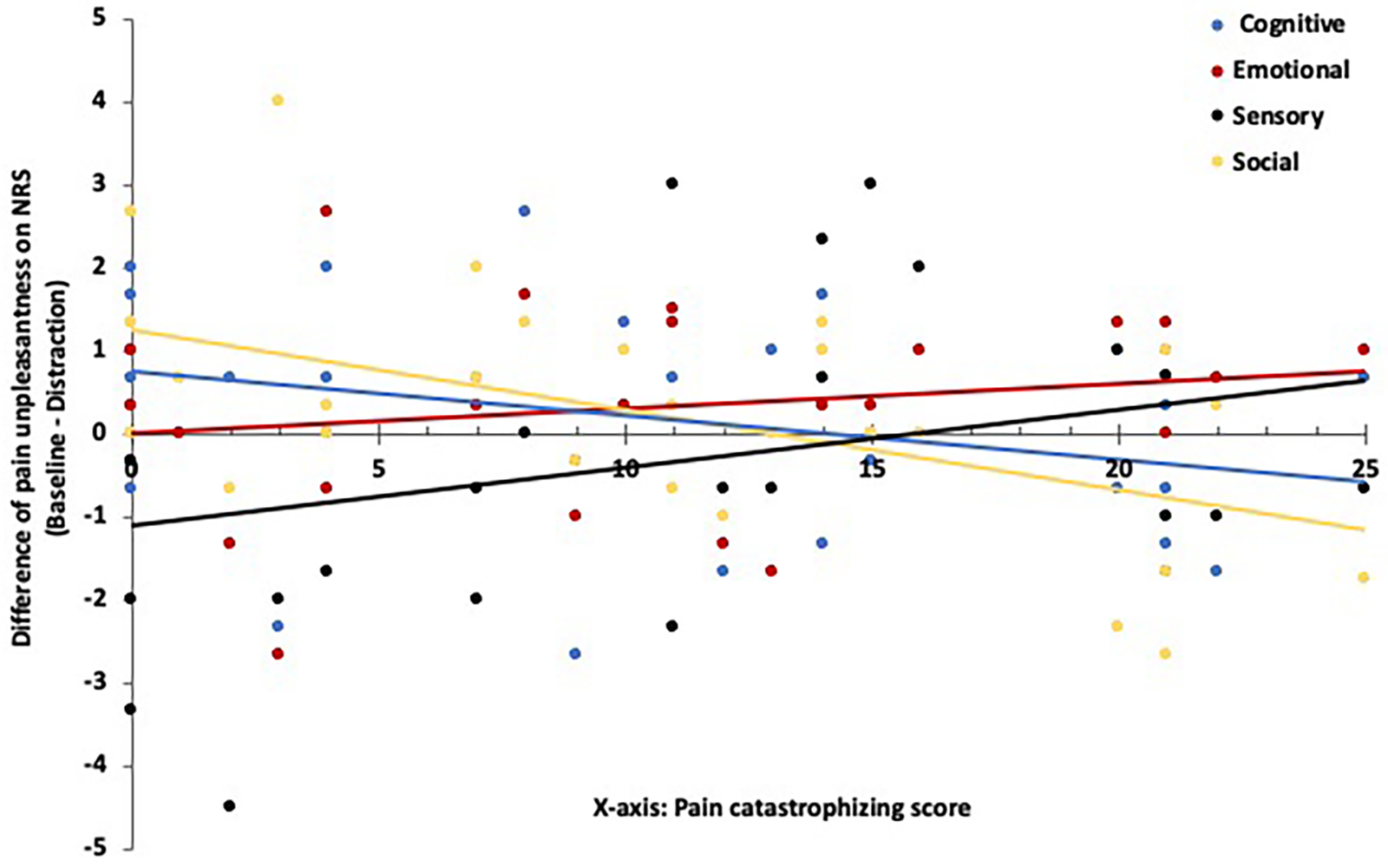
Visual presentation of the PCS's contribution to the change of pain unpleasantness in NRS scale depended on the determined distraction task and respective baseline. A positive score on the y-axis indicates a reduction in pain unpleasantness during distraction, whereas a negative score suggests an increase.

Association between PCS and pain unpleasantness during different distractions: Further analysis revealed significant slope differences in the relationship between pain unpleasantness and PCS score for social compared to sensory distraction F(1, 28) = 12.30, *p* = .002, ηp^2^ = .30) cognitive vs. sensory distraction [F(1, 28) = 6.12, *p* = .02, ηp^2^ = .18], social vs. emotional distraction [F(1, 28) = 10.30, *p* = .003, ηp^2^ = .27] and cognitive vs. emotional distraction [F(1, 28) = 9.60, *p* = .004, ηp^2^ = .26]. Differences between the other distraction tasks were not statistically significant. Figure 4 illustrates the interaction plot, demonstrating the variation in the relationship between PCS and pain unpleasantness across different distraction tasks. The correlations of PCS and changing pain unpleasantness in each distraction task were as follows: Sensory distraction (r(30) = .29, *p* = .12), cognitive [r(30)= -.31, *p* = .09], social [r(30)= -.54, *p* = .002], and emotional distraction [*r*(30) = .21, *p* = .27]. Only the correlation in social distraction was significant, suggesting a diverse effect where an increase in PCS score led to a smaller reduction or even an increase in pain unpleasantness during this distraction task.

## 4 Discussion

The objective of this study was to examine pain dimensions (intensity and unpleasantness) across four distinct distraction tasks, evaluate these differences while controlling for pain catastrophizing, and ultimately assess the relationship between pain catastrophizing and pain dimensions during these distraction tasks. Cognitive distraction was found to be more effective than other tasks in reducing pain intensity and unpleasantness. When accounting for pain catastrophizing, no notable advantage was found among the distraction tasks relative to one another. However, the relationship between PCS scores and pain intensity exhibited variability across distraction tasks. Notably, cognitive and social distractions had diverse effects compared to the sensory task, with only social distraction demonstrating a significant correlation. This correlation suggested that an elevated PCS score was associated with a reduced reduction or even an increase in pain intensity during this specific distraction task. A similar pattern was observed in pain unpleasantness, albeit with slight variations. Cognitive and social distractions exerted distinct influences compared to sensory and emotional distractions. As pain catastrophizing levels increased, participants reported reduced unpleasantness during emotional and sensory distractions. However, in the case of cognitive and social distractions, an elevated PCS score led to a diminished reduction or even an increase in pain unpleasantness. Importantly, the significant correlation between PCS scores and pain unpleasantness was solely evident in the context of social distraction. These findings emphasize the different interactions of pain catastrophizing within various distraction tasks.

Many studies have investigated the effects of distraction on pain perception, focusing on different aspects, such as experimental pain induction, age, patients with chronic pain, and healthy subjects (6, 7, 18). In two separate studies, Van Ryckeghem et al. showed that distraction was effective for healthy adults during electrically induced pain using auditory and somatosensory distraction. However, individuals who perceived the electrical stimulus as more painful did not benefit as much from distraction compared to others (11, 12). In a study by Thompson et al., distraction tasks were examined in healthy participants using thermal noxious stimuli, revealing that distraction was generally effective regardless of anxiety levels (27). The discrepancy between previous studies can be attributed to the importance of pain characteristics and their impact on pain perception during distraction tasks. The severity and the perceived threat of pain are crucial elements in the pain environment (50, 51). As the intensity of the pain stimulus increases, it becomes challenging to divert attention away from the source of pain. Additionally, using electrical stimulation to induce pain may introduce a sense of threat and further direct attention toward the pain sensation. It is important to acknowledge that relying solely on distraction may not be sufficient to reduce pain effectively, and other contributing factors should be considered. Maintaining focus on the distraction task and minimizing potential pain-related distractions is necessary for achieving optimal outcomes.

In line with the literature, low and high pain catastrophizers reacted differently to different kinds of distraction tasks (9, 15, 36). In a study that compares the effects of mindfulness and distraction on pain, Prins et al. found that in low pain catastrophizers, distraction had more benefits on pain perception than mindfulness technique (which direct attention to the stimulus in a judgmental way). However, in high pain catastrophizers, the mindfulness technique was more beneficial (9). Several studies support the present findings that in individuals with a high level of pain catastrophizing, distraction tasks are less effective (9, 15, 17). However, a recent study about the role of selective attention and pain catastrophizing showed that the cognitive distraction task was effective in high rather than low pain catastrophizers, which is in contradiction with our result (36). This discrepancy may be attributed to several factors, including the presence of motivational incentives, the moderate level of pain intensity, and the need for an adequate duration of engagement in the distraction task before initiating pain stimulation.

According to the attentional model, individuals with higher levels of pain catastrophizing tend to divert their attention to the pain-related information, making it difficult for them to disengage from the source of threat (35, 52). Cognitive and social distractions, being active forms of distraction, probably generate a cognitive load that effectively redirects attention from pain stimulus for those with lower pain catastrophizing levels. In particular, our study suggested that social distraction (incorporating both emotional and cognitive states) showed more significant benefits with lower PCS scores than with higher PCS scores. As pain catastrophizing increased, the existence of threat-related information placed a significant attentional demand, hindering disengagement from the pain stimulus. An additional explanation related to the analgesic mechanism of stress-induced analgesia (SIA) within the context of distraction tasks. This phenomenon suggests that stress may suppress pain perception (10). Individuals with low PCS are not helpless in painful situations (29), and stress could not reach a level to induce analgesia. In our study, individuals at the highest end of the PCS spectrum may still exhibit low scores of PCS, potentially inadequate to trigger these analgesic mechanisms. Activation of this mechanism requires a certain degree of stress level, as it is linked to the fight or flight response (53). Although, Kunz et al. (2016) found a mean PCS score of 13.6 (SD = ± 8.4) in pain-free individuals and 18.3 (SD = ±8.2) in young acute pain patients in the German population (50) which is similar to the PCS score observed in our study sample.

A sensory task differs from other forms of distraction in its approach. It shifts attention towards the sensation of pain, ultimately reducing discomfort and emotional distress by engaging the sensory pain processing mode rather than the emotional mode (54). This method, rooted in meditation, relaxation, and yoga, promotes a non-judgmental acceptance of pain and supports the body in managing pain and negative emotions (26, 55). Due to the bottom-up route processing, heightened pain catastrophizing directs attention more towards the sensory aspects of pain rather than the emotional, thereby suppressing negative appraisal. This mechanism has the potential to counteract pain catastrophizing and negative appraisal, mainly when the pain intensity is not excessively high. This can occur by directing attention to the present situation rather than future concerns or maintaining awareness of the pain sensation without exaggerating its actual intensity (9). Our study revealed that pain unpleasantness decreased during sensory distraction as PCS levels increased. However, it is important to highlight that these patterns were not observed in lower levels of PCS scores. This difference could potentially be attributed to the pain intensity used in the study, which may not have been sufficiently high to elicit significant effects, or the distraction task employed might not have been appealing enough to divert their attention effectively.

Finally, emotional distraction did not demonstrate changes in pain intensity with alterations in pain catastrophizing. In line with our study, Villarreal et al. reported that active cognitive distraction is superior to emotional distraction. The efficacy of emotional distraction mainly depends on the cognitive and emotional components of the task. This distraction, particularly with sound or music, relies heavily on the familiarity and recognition of the memory process based on the individual's experience (10). In our study, the sound was selected by the test leader. It could be due to the fact that the pleasure and arousal factors of the sounds may not have been sufficient to evoke a change in the affective pathway of pain. Moreover, emotional distraction led to the reduction of pain unpleasantness with increasing pain catastrophizing. An alternative explanation could be related to the characteristics of the participants themselves. Individuals with higher levels of PCS may tend to be more emotionally oriented compared to those with lower levels of PCS. As a result, engaging in activities such as listening to music may have a stronger impact on their perception, serving as an effective form of emotional distraction. Individuals with lower levels of PCS may evaluate pain more rationally, wherein emotional distractions have less influence on their perceived degree of unpleasantness.

The present study has some limitations. First, chronic patients have a unique profile for pain perception. Each patient could adjust the connection between distraction and pain catastrophizing differently due to mood swings, cognitive alteration, and emotional instability (2). In order to limit these secondary variables, this study selected only pain-free individuals. Second, the PCS scores in our study, which ranged from 0 to 25. This range falls within the medium score range when considering the maximum score of 52. It is possible that our study may not have fully captured the characteristics and experiences of individuals with high levels of PCS. A wider range of PCS scores, particularly at the higher end, may provide more insights into the relationship between pain catastrophizing and the effectiveness of sensory distraction. Last, in this study, we did not manipulate the workload of distraction tasks (56–58). Workload manipulation as an influencing factor of the distraction's efficacy has to be considered in further studies.

## 5 Conclusion

Our findings suggest a non-uniform interaction between PCS and both pain intensity and unpleasantness during various distraction tasks. Depending on the characteristics of the distraction task, distinct relationships may be observed. Specifically, social and cognitive distractions exhibited different relationships than sensory distractions concerning PCS and pain dimensions. In particular, social distraction, marked by emotional and cognitive states, proves advantageous with lower PCS scores; however, this benefit diminishes as PCS scores increase.

## 6 Clinical implication

Persistent exposure to pain can result in the formation of memories and alterations at various levels of the pain system. Classical and instrumental conditioning mechanisms may play a significant role in individuals experiencing pain. Consequently, therapeutic interventions aimed at managing pain should encompass identifying and addressing maladaptive pain behaviors, reducing pain, and cultivating positive expectations to disrupt the cycle of pain behavior. In summary, the findings of our study indicate that the application of distraction as a pain management strategy should not be uniformly applied to all populations. Based on the biopsychosocial model, multiple factors need to be considered to achieve the optimal effects of distraction techniques on pain perception. The level of pain catastrophizing, functioning as a cognitive factor, can impact pain perception depending on the type of distraction tasks.

## Data Availability

The raw data supporting the conclusions of this article will be made available by the authors, without undue reservation.
